# Chemical Composition and Possible *in Vitro *Phytotoxic Activity of *Helichrsyum italicum* (Roth) Don ssp. *italicum*

**DOI:** 10.3390/molecules16097725

**Published:** 2011-09-08

**Authors:** Emilia Mancini, Laura De Martino, Aurelio Marandino, Maria Rosa Scognamiglio, Vincenzo De Feo

**Affiliations:** 1 Dipartimento di Scienze Farmaceutiche e Biomediche, Università degli Studi di Salerno, via Ponte Don Melillo, 84084 Fisciano (Salerno), Italy; Email: emancini@unisa.it (E.M.); ldemartino@unisa.it (L.D.M.); aureliomarandino@libero.it (A.M.); 2 Dipartimento di Ingegneria Industriale, Università degli Studi di Salerno, via Ponte Don Melillo, 84084 Fisciano (Salerno), Italy; Email: mrscogna@unisa.it (M.R.S.)

**Keywords:** *Helichrysum italicum *(Roth) Don ssp. *italicum*, essential oil, phytotoxicity

## Abstract

The chemical composition of the essential oil of *Helichrysum italicum *(Roth) Don ssp*. italicum*, collected in the National Park of Cilento and Diano Valley, Southern Italy, was studied by means of GC and GC/MS. Forty four compounds of 45 constituents were identified in the oil, mainly oxygenated sesquiterpenes. The essential oil was evaluated for its potential *in vitro *phytotoxic activity against germination and early radicle elongation of radish and garden cress. The radicle elongation of radish was significantly inhibited at the highest doses tested, while germination of both seeds was not affected.

## 1. Introduction

The genus *Helichrysum* (family Asteraceae, tribe Inuleae) is a very large genus including 600 species widespread diffused throughout the World. *Helichrysum *species are xerophytes which are distributed from the lower-meso-Mediterranean to the lower-sub-humid bioclimatic environments, growing at a wide range of altitudes from the sea level up to 1700 m a.s.l., preferably on sandy or loamy soils [1]. Coastal sand dunes are structures of Mediterranean area which protect the coast by absorbing energy from wind, wave action, and host ecosystems made up of colonizer species. The typical sand dune communities are a portion of the larger “macchia”, that becomes “garigue” in its degradated state. In this area *Helichrysum* species can create some dense monophytic communities.

Several studies reported a high degree of polymorphism in *Helichrysum *[2], which produces different ecotypes [3]. Even though this large genus has been extensively studied, the chemical variations and the genetic relationships within the species still remain unclear [4].

Almost 25 species of *Helichrysum *are native of Mediterranean area, eight belonging to Italian Flora. Italian *Helichrysum italicum* comprises two subspecies, *H. italicum* (Roth) Don ssp. *italicum* and *H. italicum* ssp. *microphyllum* (Willd.) Nyman [3]. *H. italicum* ssp. *italicum*, is a small aromatic shrub, up to 40–50 cm high, with yellow flowers, growing on dry cliffs and sandy soil [5].

Dried inflorescences of this plant are traditionally used as a moth antifeedant, in the area of the National Park of Cilento and Diano Valley (Campania, Southern Italy); a decoction of flowering tops is used for fumigations in the treatment of asthma [6].

Several studies on the composition of essential oil of *H. italicum* have been reported in literature. Morone-Fortunato and co-workers [4] reported three different chemotypes for the essential oils of *H. italicum* ssp. *italicum*: (I) a genotype rich in nerol and its esters; (II) a genotype with a dominance of α- and β-selinene; (III) a genotype with high amounts of γ-curcumene. Essential oils from *H. italicum* ssp. *italicum*, characterized by high amounts of monoterpenoids such as neryl acetate, neryl propanoate and α-pinene, have been described by Bianchini and co-workers [7] and Paolini and co-workers [8]. An oil rich in geraniol and geranyl acetate has been reported for plants collected in Greece [7,9]. Other studies reported populations of *H. italicum* ssp. *italicum *characterized by major amounts of sesquiterpenes [7].

Metabolites isolated from *H. italicum*, and especially its volatile fraction, have been found to display pharmacological properties, such as anti-inflammatory [10,11], antiallergic, antibacterial, antifungal [12,13,14,15,16], antioxidant [10,17] and antiviral activity [11,18]. The antimicrobial and insecticidal effects of the essential oil, have been also reported [12,13,14,15,16,19]; no reports on the possible phytotoxic activity of the secondary metabolites of the plant have been reported.

In continuation of our studies on the chemistry of essential oils from Mediterranean flora and their possible *in vitro *phytotoxic activity [20], we analyzed the chemical composition of the essential oil of *Helichrysum italicum* (Roth) Don ssp. *italicum*, collected in the National Park of Cilento and Diano Valley, examining also its possible phytotoxic effects against germination and early radicle elongation of *Raphanus sativus* L. (radish) and *Lepidium sativum* L. (garden cress).

## 2. Results and Discussion

### 2.1. Chemical Composition of the Essential Oil

Hydrodistillation yielded 0.02% of a pale yellow oil (on a dry mass basis)*. *The volatile components of the essential oil of *H. italicum* ssp. *italicum* and their percentages are shown in Table 1, according to their elution order on a HP-5 MS column. Fourty-four compounds were identified, accounting for 90.0% of the total oil. Sesquiterpenes represented 74.3% of the total oil, while the monoterpenoid fraction accounted only for 1.1%. Oxygenated sesquiterpenes (73.6%) prevailed, being the major constituents *iso*-italicene epoxide (16.8%), β-costol (7.5%) and (*Z*)-α-*trans*-bergamotol (4.7%).

**Table 1:** Essential oil composition (%) of *Helichrysum italicum* spp. *italicum* growing wild in the National Park of Cilento and Diano Valley.

Ri ^a^	Ri ^b^	Compound	Identification ^c^	%
**Monoterpenoid hydrocarbons**				**1.1**
1165	1652	Menthol	1,2,3	1.1
**Sesquiterpenoid hydrocarbons**				**0.7**
1408	1666	(*Z*)-Caryophyllene	1,2	0.1
1504	1743	α-Bisabolene	1,2	0.6
**Oxygenated sesquiterpenoids**				**73.6**
1510	2022	*iso*-Italicene epoxide	1,2	16.8
1551		*cis*-Cadinene ether	1,2	0.5
1565	2050	(*E*)-Nerolidol	1,2	0.5
1568	2001	Caryophyllenyl alcohol	1,2	1.4
1581	1871	Neryl isovalerate	1,2	0.5
1585	2008	Caryophyllene oxide	1,2	1.4
1600	2108	Guaiol	1,2	2.3
1609		*cis*-Isolongifolanone	1,2	1.4
1615		Isolongifolan-7-α-ol	1,2	0.9
1617	2127	10-*epi-*γ-Eudesmol	1,2	0.9
1620		(*Z*)-Bisabolol-11-ol	1,2	1.4
1627	2250	1,10-di-*epi*-Cubenol	1,2	2.3
1635	2185	γ-Eudesmol	1,2	1.4
1641		*allo*-Aromadedrene epoxide	1,2	2.3
1648	2258	β-Eudesmol	1,2	1.4
1655	2250	α-Eudesmol	1,2	0.9
1657	2274	4α-H-Eudesm-11-en-4-ol	1,2	1.4
1662		α-Betulenol	1,2	1.8
1668		(*E*)-Bisabol-11-ol	1,2	0.5
1670	2357	14-Hydroxy-9-*epi*-(*E*)-caryophyllene	1,2	0.5
1675	2170	β-Bisabolol	1,2	0.5
1678		(*Z*)-Nerolidyl acetate	1,2	2.3
1682	2400	*epi-*α-Bisabolol	1,2	0.9
1688		8-Cedren-13-ol	1,2	4.2
1697	2247	(*Z*)-α-*trans*-Bergamotol		4.7
1699	2273	Selin-7(11)-en-4-ol		1.4
1704		Amorpha-4,9-dien-14-al		2.8
1709		Sesquiterpene	1,2	1.4
1711		(2*E*,6*Z*)-Farnesal	1,2	1.4
1717		(*E*)-Nerolidyl acetate	1,2	0.9
1726		Guaiol acetate	1,2	0.5
1750		6*S*,7*R*-Bisabolone	1,2	2.3
1758	2580	2,6-Bisaboladien-12-ol	1,2	2.3
1768	2606	β-Costol	1,2	7.5
**Hydrocarbons**				**11.4**
1391	1433	Tetradecene	1,2	0.7
1499	1500	Pentadecane	1,2	0.9
1592	1654	Hexadecene	1,2	9.8
**Fatty acids**				**0.9**
1588		Octanedioic acid, diethyl ester	1,2	0.9
**Others **				**1.4**
1572	2077	*n*-Tridecanol	1,2	1.4

^a^ Kovats retention index on HP-5 MS column; ^b^ Kovats retention index on HP Innowax; ^c^ 1 = Kovats retention index, 2 = mass spectrum, 3 = coinjection with authentic compound.

The composition of our sample appears to be different from the composition of Spanish oil of *H. italicum* ssp*. serotinum*, in which the major components were guaiol (8.9%), nerol (7.0%) and β-caryophyllene (6.0%) [21].

Bianchini and co-workers [22] reported that the essential oils obtained from Corsican and Tuscan *H. italicum *ssp. *italicum *exhibited two different compositions. Corsican oils were characterized by the prevalence of oxygenated compounds: neryl acetate (major compound), neryl propionate, nerol, acyclic ketones and β-diketones. Tuscan oils were found to exhibit higher contents of hydrocarbons (α-pinene, β-caryophyllene, α- and β-selinene) [22]. Neryl acetate was also reported as one of the main components of the oil of *H. italicum *ssp. *italicum *from North America [23] and in the oil of *H. italicum *from South Croatia [13]. Nerol and its esters have also been found as the main components of the essential oil from flowers of some genotypes of *H. italicum* ssp. *microphyllum* [24].

Morone-Fortunato and co-workers [4] reported the chemical compositions of 20 native *Helichrysum italicum* ssp. *italicum* genotypes. Some of them can be distinguished by the high content of sesquiterpenoids (from 61.6% to 91.3%), such as γ-curcumene, α-selinene and rosifoliol.

Usai and co-workers [25] reported the chemical composition and variation of the essential oil of sardininan *H. italicum* ssp. *microphyllum* under different weather conditions, from their vegetative period to post-blooming time. Components present in highest percentage were neryl acetate (17.6**–**35.6%), nerol (3.7**–**14.4%) and eudesmen-5-en-11-ol (6.4**–**23.5%). Among them, the neryl acetate percentage decreased when 5-eudesmen-11-ol percentage increased. Furthermore, Bianchini and coworkers [26] reported the presence of eudesm-5-en-11-ol in essential oil from this species.

Tundis and co-workers [27] reported the biovariability of *H. italicum* (Roth) Don grown wild in Calabria and Sardinia. Also in this case, neryl acetate was one of the main compounds and it was considered as a chemical marker. Blaževic and coworkers [28] investigated differences in essential oils of *H. italicum*, collected in different stage of development and different locations along Adriatic coast.

Marongiu and coworkers [29] reported the analysis of the leaves and flowers of *Helichrysum italicum* (Roth) Don ssp. *microphyllum* (Willd.) Nyman by supercritical fluid extraction and compared the composition of the oils obtained with this technique with oils obtained by hydrodistillation.

Differences in the composition between our sample and those reported in literature can be explained by various factors, e.g., the harvest time, local, climatic, geographical, seasonal factors [4].

Generally, chemotypes can determine “biochemical varieties” or “physiological forms” in botanical species, each of which has a specific enzymatic equipment. These species are genetically codified and direct their biosynthesis to the preferential formation of a definite compound. The characterization of habitat is of fundamental importance to understand species distribution. In a definite geographical area, the factors that weight heavily on chemotypes differentiation are mainly related to intrinsic factors such as sexual polymorphism or genetic mechanism, but for the essences, environmental conditions are able to influence biosynthetic pathway.

The essential oil was evaluated for its phytotoxic activity against germination and initial radicle elongation of radish and garden cress, two species frequently utilized in biological assays [30]. The germination of radish and garden cress did not appear significantly sensitive to the essential oil (Table 2). At doses ranging between of 2.5 and 0.25 μg/mL the essential oil significantly inhibited the radicle elongation of radish of about 30% (Table 3). The roots were probably more sensitive than shoots to the phytotoxic activity of the oil: the process of germination was active while the oil probably affected the elongation process.

**Table 2:** Biological activity of essential oil of *Helichrysum italicum* spp. *italicum* against germination of *Raphanus sativus *(radish) and *Lepidium sativum *(garden cress), 120 h after sowing. Results are the mean ± standard deviation (SD) of three experiments.

	*Helichrysum italicum *ssp. *italicum *
***Raphanus sativus***	**Germinated seeds ± SD**
**Control**	9.3 ± 1.0
**2.5** μg/mL	9.7 ± 0.6
**1.25** μg/mL	9.0 ± 0.0
**0.625** μg/mL	8.0 ± 1.0
**0.25** μg/mL	9.0 ± 1.0
**0.125** μg/mL	9.3 ± 2.1
**0.06** μg/mL	8.3 ± 0.6
***Lepidium sativum ***	**Germinated seeds ± SD**
**Control**	8.7 ± 1.2
**2.5** μg/mL	8.7 ± 0.6
**1.25** μg/mL	9.7 ± 0.6
**0.625** μg/mL	9.7 ± 0.6
**0.25** μg/mL	8.7 ± 0.6
**0.125** μg/mL	9.7 ± 0.6
**0.06** μg/mL	9.3 ± 1.2

Such activity of the essential oil could help to explain the ecological role of genus *Helichrysum *in the Mediterranean area. In general, several studies have documented that the oil from aromatic plants and volatile terpenes are potent inhibitors of seed germination and radicle elongation. For example, Muller and co-workers [31] demonstrated that volatile oils from *Salvia leucophylla* Greene and *Artemisia californica *Less. reduced the growth of associated plants, thus resulting in characteristic vegetational patterning [32]. Moreover, phytotoxicity of volatile oils and terpenes also has been implicated as one of the possible reasons for successful colonization by invasive weeds [32].

**Table 3:** Biological activity of essential oil of *Helichrysum italicum* spp. *italicum* against radicle elongation of *Raphanus sativus *(radish) and *Lepidium sativum *(garden cress), 120 h after sowing. Data are expressed in cm. Results are the mean ± standard deviation (SD) of three experiments.

	*Helichrysum italicum *ssp. *italicum *
***Raphanus sativus***	**Radicle length ** **± SD**
**Control**	9.0 ± 4.6
**2.5** μg/mL	6.4 ± 3.5 **
**1.25** μg/mL	6.5 ± 3.3 *
**0.625** μg/mL	7.6 ± 3.7
**0.25** μg/mL	6.6 ± 4.7 *
**0.125** μg/mL	8.6 ± 4.0
**0.06** μg/mL	8.4 ± 4.1
***Lepidium sativum ***	**Radicle length ** **± SD**
**Control**	7.5 ± 2.8
**2.5** μg/mL	6.2 ± 2.6
**1.25** μg/mL	6.4 ± 1.6
**0.625** μg/mL	6.2 ± 3.3
**0.25** μg/mL	6.7 ± 3.0
**0.125** μg/mL	6.5 ± 3.3
**0.06** μg/mL	7.0 ± 2.5

Note: * p < 0.05; ** p < 0.01; *** p < 0.001 *vs. *positive control.

Although the mode of inhibitory action of essential oils against germination still remains unclear, volatile oils and terpenoids are known to inhibit cell division and induce structural breaks and decomposition in roots [33,34,35,36]. Both monoterpenoids and sesquiterpenoids appear to be involved in these biological activities. Recently, the ecological role of sesquiterpenoids was reviewed and it was demonstrated that they inhibit the seedling growth of associated native vegetation, and thus help in successful invasion in the introduced sites [37].

## 3. Experimental

### 3.1. Plant Material

Aerial parts of *Helichrysum italicum *(Roth) Donssp. *italicum *were gathered in Marina di Camerota (National Park of Cilento and Diano Valley, Southern Italy). The plant was collected during full blooming, in May 2010. Plant was identified by V. De Feo. A voucher specimen of plant, labelled DF47/2010, was stored in the Herbarium of the Medical Botany Chair at Salerno University.

### 3.2. Isolation of the Volatile Components

One hundred grams of freshly aerial parts of sample were ground in a Waring blender and then subjected to hydrodistillation for 3 h according to the standard procedure described in the European Pharmacopoeia [38]. The oil was solubilised in *n*-hexane, filtered over anhydrous sodium sulphate and stored under N_2_ at +4 °C in the dark until tested and analyzed.

### 3.3. Gas Chromatography

Analytical gas chromatography was carried out on a Perkin-Elmer Sigma-115 gas chromatograph equipped with a FID and a data handling processor. The separation was achieved using a HP-5 MS fused-silica capillary column (30 m × 0.25 mm i.d., 0.25 μm film thickness). Column temperature: 40 °C, with 5 min initial hold, and then to 270 °C at 2 °C/min, 270 °C (20 min); injection mode splitless (1 μL of a 1:1,000 *n*-pentane solution). Injector and detector temperatures were 250 °C and 290 °C, respectively. Analysis was also run by using a fused silica HP Innowax polyethylenglycol capillary column (50 m × 0.20 mm i.d., 0.25 μm film thickness). In both cases, helium was used as carrier gas (1.0 mL/min).

### 3.4. Gas Chromatography-Mass Spectrometry

Analysis was performed on an Agilent 6,850 Ser. II apparatus, fitted with a fused silica DB-5 capillary column (30 m × 0.25 mm i.d., 0.33 μm film thickness), coupled to an Agilent Mass Selective Detector MSD 5973; ionization energy voltage 70 eV; electron multiplier voltage energy 2,000 V. Mass spectra were scanned in the range 40–500 amu, scan time 5 scans/s. Gas chromatographic conditions were as reported in the previous paragraph; transfer line temperature, 295 °C.

### 3.5. Identification of Components

Most constituents were identified by gas chromatography by comparison of their Kovats retention indices (Ri) with either those of the literature [39,40] or with those of authentic compounds available in our laboratories. The Kovats retention indices were determined in relation to a homologous series of *n*-alkanes (C_10_–C_35_) under the same operating conditions. Further identification was made by comparison of their mass spectra on both columns with either those stored in NIST 02 and Wiley 275 libraries or with mass spectra from the literature [39,41] and a home made library. Components relative concentrations were obtained by peak area normalization. No response factors were calculated.

### 3.6. Biological Assay

A bioassay based on germination and subsequent radicle growth was used to study the phytotoxic effects of the essential oil of *H. italicum* ssp. *italicum* on seeds of *Raphanus sativus *L. cv. “*Saxa*” (radish) and *Lepidium sativum *L. (garden cress). The seeds were purchased from Blumen srl (Piacenza, Italy). The seeds were surface sterilized in 95% ethanol for 15 s and sown in Petri dishes (Ø = 90 mm), containing five layers of Whatman filter paper, impregnated with distilled water (7 mL, control) or tested solution of the essential oil (7 mL), at the different assayed doses. The germination conditions were 20 ± 1 °C, with natural photoperiod. The essential oil, in water–acetone mixture (99.5:0.5), was assayed at the doses of 2.5, 1.25, 0.625, 0.25, 0.125 and 0.062 μg/mL. Controls performed with water-acetone mixture alone showed no appreciable differences in comparison with controls in water alone. Seed germination was observed directly in Petri dishes, each 24 h. A seed was considered germinated when the protrusion of the root became evident [42]. After 120 h (on the fifth day), the effects on radicle elongation were measured in cm. Each determination was repeated three times, using Petri dishes containing 10 seeds each. Data are expressed as the mean ± SD for both germination and radicle elongation. Data were ordered in homogeneous sets, and the Student’s *t* test of independence was applied [43].

## 4. Conclusions

Considering the remarkable variations reported for the essential oil of *Helichrysum italicum *ssp. *italicum *from different localities, it was of interest to further examine its chemical polymorphism. Our data might be a valuable chemotaxonomic marker for further classification and subdivision of the genus *Helichrysum.* Our *in vitro *experiments on the essential oil from *H. italicum *ssp*. italicum *show that its essential oil was active against radicle elongation of radish*. *The phytotoxic activity was probably due to the presence of bioactive sesquiterpenes [12,44], which can suggest helpful models for lead compounds in the development of new herbicides [45].
